# Poisoning due to *Arisaema triphyllum* Ingestion

**DOI:** 10.5005/jp-journals-10071-23171

**Published:** 2019-05

**Authors:** Dhiraj Ramdas Jadhav, Ramesh Gugloth

**Affiliations:** 1,2 Department of Emergency Medicine, Dr DY Patil Medical College, Pune, Maharashtra, India

**Keywords:** Airway compromise, *Arisaema triphyllum*, Hypersalivation

## Abstract

Plants of arum family are beautiful and attractive but, at the same time, they are poisonous. The toxic effects are due to calcium oxalate crystals. The toxicity ranges from minor oral cavity edema to lethal airway obstruction.

**How to cite this article:** Jadhav DR, Gugloth R. Poisoning due to *Arisaema triphyllum* Ingestion. Indian J Crit Care Med 2019;23(5):242–243.

## INTRODUCTION

Oxalate containing plants are widely present as different species like Anthurium (*Anthurium* species) Arum, Araceae (*Arisaema* species), Caladium (*Caladium bicolor*), Calla lily (*Zantedeschia* species), Chinese evergreen (*Aglaonema* species), Dieffenbachia etc.^1^

Calcium oxalate containing plants like Dieffenbachia are known for their irritant properties since last 200 years. They were used to treat gout, impotence, and its frigidity was also used to punish slaves. Today, they are admired for their ornamental beauty, so they are present in abundance at public places.^2^ We are presenting a case of *Arisaema triphyllum* poisoning with oral cavity and airway edema.

## CASE REPORT

A 4-year-old child was brought to emergency department (ED) with complaints of excessive salivation and inability to speak since 2 hours after consuming roots of unknown plant while playing in the garden. He also had pricking sensation in throat, chest discomfort, and shortness of breath. There was no history of vomiting and abdominal pain. General physical examination revealed the pulse rate of 92 beats per minute, blood pressure of 114/76 mm Hg, SPO_2_ of 96% on room air, and respiratory rate (RR) of 18 breaths per minute. Examination of oral cavity showed excessive salivation, congestion of posterior pharyngeal wall, and swelling of uvula. Respiratory system examination revealed conducted sounds while other systemic examinations were normal. The diagnosis of *Arisaema triphyllum* ingestion was confirmed after child has identified the plant brought by his father. The child was treated with IV fluids, intramuscular injection adrenaline 0.3 mL (1:1000 dilution) for possible airway edema and obstruction. Pain relief was obtained initially with cold water and later with tablet paracetamol. Patient was continuously monitored for possible worsening due to airway compromise. After 24 hours of observation, patient was discharged.

## DISCUSSION

*Arisaema triphyllum,* also known as jack in the pulpit, Indian turnip, bog onion, and brown dragon, belongs to family Aracea or Arums.^3^ The toxicity of *Arisaema triphyllum* is due to calcium oxalate crystals, which are mainly present in stem, leaves, and roots. The roots are considered as the most toxic part.^4^

Calcium oxalates are needle-like crystals, which when ingested, may pierce the mouth, throat, and digestive tract as they pass through and cause an intense discomfort, produce pain and edema. It also affects conjunctiva and skin when it comes in contact. Edema primarily is due to direct trauma from the needle-like crystals but bradykinins also play some role in edema formation.^5,6^

**Fig. 1:**
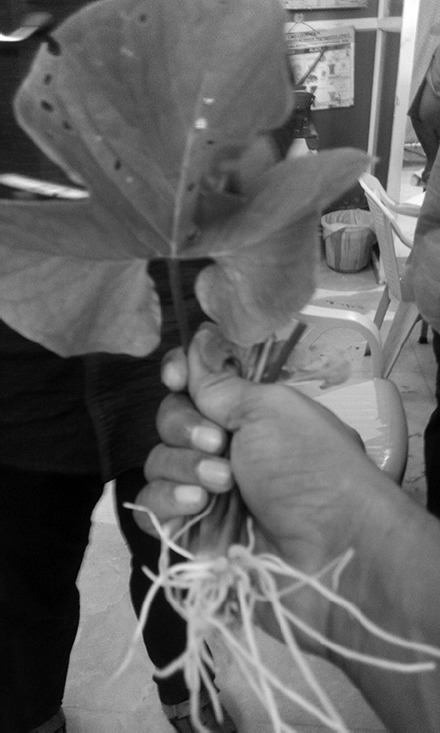
Philodendron leaves

Majority of cases are among children younger than five years. Most cases are self-limiting, and morbidity and mortality is rare. But small dose of oxalate toxin is enough to cause intense sensations of burning in the mouth and throat, swelling, hypersalivation, and choking. The airway edema and obstruction can possibly be lethal.

There is no diagnostic test to confirm diagnosis. Only high index of suspicion and clear history of ingestion often gives clue for diagnosis. Treatment is often symptomatic in most of the cases, but emergency physician must be aware of potentially threatening airway and should prepare for it. One case of infant fatality is reported due to obstructed airway after ingestion of Dieffenbachia plant and another child death is reported due to vagotonia attributed to esophageal lesion caused by ingestion of philodendron leaves (Fig. 1), which contains calcium oxalate as a principle toxin similar to *Arisaema triphyllum*.^7,8^

## CONCLUSION

Though arum poisoning is self-limiting most of the time, it can be lethal so emergency physician should always be ready for difficult airway.
